# Progressive remodeling of structural networks following surgery for operculo-insular epilepsy

**DOI:** 10.3389/fneur.2024.1400601

**Published:** 2024-07-31

**Authors:** Sami Obaid, Guido I. Guberman, Etienne St-Onge, Emma Campbell, Manon Edde, Layton Lamsam, Alain Bouthillier, Alexander G. Weil, Alessandro Daducci, François Rheault, Dang K. Nguyen, Maxime Descoteaux

**Affiliations:** ^1^Department of Neurosciences, University of Montreal, Montreal, QC, Canada; ^2^University of Montreal Hospital Research Center (CRCHUM), Montreal, QC, Canada; ^3^Division of Neurosurgery, Department of Surgery, University of Montreal Hospital Center (CHUM), Montreal, QC, Canada; ^4^Sherbrooke Connectivity Imaging Lab (SCIL), Sherbrooke University, Sherbrooke, QC, Canada; ^5^Department of Neurology and Neurosurgery, Faculty of Medicine, McGill University, Montreal, QC, Canada; ^6^Department of Computer Science and Engineering, Université du Québec en Outaouais, Montreal, QC, Canada; ^7^Department of Psychology, University of Montreal, Montreal, QC, Canada; ^8^Department of Neurosurgery, Yale School of Medicine, Yale University, New Haven, CT, United States; ^9^Division of Pediatric Neurosurgery, Department of Surgery, Sainte Justine Hospital, University of Montreal, Montreal, QC, Canada; ^10^Department of Computer Science, University of Verona, Verona, Italy; ^11^Medical Imaging and Neuroimaging (MINi) Lab, Sherbrooke University, Sherbrooke, QC, Canada; ^12^Division of Neurology, University of Montreal Hospital Center (CHUM), Montreal, QC, Canada

**Keywords:** insula, epilepsy, epilepsy surgery, plasticity, tractography, connectome

## Abstract

**Introduction:**

Operculo-insular epilepsy (OIE) is a rare condition amenable to surgery in well-selected cases. Despite the high rate of neurological complications associated with OIE surgery, most postoperative deficits recover fully and rapidly. We provide insights into this peculiar pattern of functional recovery by investigating the longitudinal reorganization of structural networks after surgery for OIE in 10 patients.

**Methods:**

Structural T1 and diffusion-weighted MRIs were performed before surgery (t_0_) and at 6 months (t_1_) and 12 months (t_2_) postoperatively. These images were processed with an original, comprehensive structural connectivity pipeline. Using our method, we performed comparisons between the t_0_ and t_1_ timepoints and between the t_1_ and t_2_ timepoints to characterize the progressive structural remodeling.

**Results:**

We found a widespread pattern of postoperative changes primarily in the surgical hemisphere, most of which consisted of reductions in connectivity strength (CS) and regional graph theoretic measures (rGTM) that reflect local connectivity. We also observed increases in CS and rGTMs predominantly in regions located near the resection cavity and in the contralateral healthy hemisphere. Finally, most structural changes arose in the first six months following surgery (i.e., between t_0_ and t_1_).

**Discussion:**

To our knowledge, this study provides the first description of postoperative structural connectivity changes following surgery for OIE. The ipsilateral reductions in connectivity unveiled by our analysis may result from the reversal of seizure-related structural alterations following postoperative seizure control. Moreover, the strengthening of connections in peri-resection areas and in the contralateral hemisphere may be compatible with compensatory structural plasticity, a process that could contribute to the recovery of functions seen following operculo-insular resections for focal epilepsy.

## 1 Introduction

Operculo-insular epilepsy (OIE) is a rare form of focal epilepsy that can mimic frontal, temporal or parietal lobe epilepsy (1, 2). Despite the difficulty in diagnosing it (1–5), detection of OIE has considerably increased in the past 20 years (1, 5–8). This heightened awareness has now brought OIE to recognition as a significant cause of drug-resistant epilepsy (DRE) (1, 5, 8, 9) for which surgical treatment is considered in well-selected cases (10–15). Due to improvements in imaging investigations, the widespread adoption of stereotactic encephalography (SEEG) and advancements in microsurgical techniques, resective surgery has become effective in controlling seizures originating from the operculo-insular region, with seizure freedom rates ranging from 60 to 80% (12, 15–27). However, despite its proven efficacy, surgery for OIE is associated with a risk of postoperative neurological complications surpassing 40% (15, 22). Yet, these safety concerns are offset by the transient course of most deficits in which the majority of patients recover fully and rapidly (15, 22). While this peculiar pattern of recovery contributes to the favorable outcome following surgery for OIE, the driving process behind the functional improvement remains unknown.

In recent years, diffusion MRI (dMRI) and tractography have been useful in assessing cross-sectional changes of white matter architecture in various types of focal epilepsy, including OIE (3, 28–33). These noninvasive approaches also lend themselves favorably to the longitudinal evaluation of white matter tracts. They have been used to investigate the progressive remodeling of specific bundles following surgery for temporal and extra-temporal epilepsies (34–39) and, in some series, to evaluate the correlation between these postoperative plastic changes and functional recovery (34, 37, 38). Some studies have specifically assessed the pattern of tractography-derived structural connectivity reorganization following focal epilepsy surgery, revealing postoperative compensatory increases in connectivity strength (CS) within unresected healthy brain regions, both ipsilateral and contralateral to the resection (34, 40). While these analyses added to our understanding of postoperative recovery in extra-insular epilepsies, no studies have looked at the longitudinal changes in white matter structure following surgery for OIE.

The aim of the study was to exploit tractography to investigate, for the first time, the longitudinal changes of structural connectivity at two predefined timepoints following surgery for OIE. To do so, we used a comprehensive pipeline that incorporates state-of-the-art tractography algorithms to derive structural connectivity networks. Our approach optimizes the reconstruction of anatomically reliable streamlines and generates valid structural connectomes. We built whole-brain tractograms using surface-enhanced tractography (SET) (41), a tracking algorithm that represents crossing fibers within voxels (42–44) and favors better cortical coverage (41, 45). Moreover, we employed Convex Optimization Modeling for Micro-structure Informed Tractography (COMMIT) to estimate the effective cross-sectional area of the axons along each streamline (46–48), a measure that can be used as a quantitative marker of CS (46–49); this weighting method allows characterization of the microstructure of underlying white matter fibers more accurately than the commonly used but contentious streamline-count (46–48, 50). Using our approach, we explore the progressive structural changes that may provide insights into the striking functional recovery commonly seen following surgery for OIE.

## 2 Materials and methods

### 2.1 Participants

We studied 10 patients with long-standing OIE (eight females; 32 ± 8 years; 18-48 years; right-sided epileptic focus in six patients) treated at the University of Montreal Hospital Center. All patients underwent a comprehensive assessment including a neurological history and examination, a neuropsychological evaluation, prolonged scalp-EEG video recordings and epilepsy protocol structural MRI scans at 3 T (T1, T2 and Fluid-attenuated inversion recovery sequences). The epileptic focus included the insula and the frontal, temporal and/or parietal operculum in nine subjects and was confined to the insula in one subject. Only three patients exhibited a small focal cortical dysplasia within the operculo-insular region on MRI. To better define the seizure origin, magnetoencephalography and intracranial EEG recordings were performed in seven and nine patients, respectively. Patients with tumoral or vascular lesions were excluded.

In addition to standard MRI sequences, high-resolution diffusion and T1-weighted (T1w) images were acquired before surgery and at two timepoints following surgery. All patients underwent a partial or subtotal open insulectomy with or without an operculectomy (10–12) carried out by a single surgeon. A trans-opercular subpial approach was performed when the operculum was involved in the epileptic focus (nine patients) while an operculum-sparing trans-sylvian approach was performed when the onset was restricted to the insula (one patient) (10–12). Surgery resulted in a favorable seizure outcome in all cases (Engel class I in eight patients and class II in two patients at last follow-up; mean follow-up time 39.6 ± 11.4 months), which confirmed that the epileptic focus was localized within the operculo-insular region. Five patients developed a postoperative neurological deficit, all of which recovered within six months. Demographics and clinical data of all included patients are listed in Table 1.

**Table 1:** Demographic and clinical information of included patients.

Patient no.	Sex	Age at surgery	Age of onset of epilepsy	Duration of epilepsy	Resection	Side of surgery	MRI: operculo-insular region	Baseline scan to surgery (months)	Surgery to first postoperative scan (months)	Surgery to second postoperative scan (months)	Follow-up duration (months)	Seizure outcome (Engel)	Postoperative deficit	Delay to recovery (months)
1	M	35	30	5	Insula (subtotal) + Fop	L	FCD	0.5	5.3	13.4	36	IIB	Aphasia	1
2	M	37	27	10	Anterior insula + Fop	R	N	6.9	6.1	12.5	60	IA	None	NA
3	F	27	9	18	Anterior insula + Fop	R	N	2.4	7	13.2	36	IA	None	NA
4	F	38	5	33	Anterior insula + FPop	L	N	3	6.2	11.8	48	IA	Contralateral hemiparesis	6
5	F	35	22	13	Posterior insula + TPop	R	N	2.7	4.97	12.9	36	IIA	Contralateral hemihypesthesia	3
6	F	33	4	29	Superior insula + FPop	L	FCD	12	7	13.8	48	IA	Aphasia	4
7	F	35	21	14	Anterior insula + Fop	R	N	1.7	8.1	14.7	36	IA	None	NA
8	F	48	12	36	Superior insula + FPop	R	FCD	7.6	6.5	12.7	48	IA	None	NA
9	F	18	10	8	Anterior insula	L	N	10.2	5.7	11.7	24	IA	None	NA
10	F	22	10	12	Insula (subtotal) + FPop	R	N	6.8	5.4	10.4	24	IA	Contralateral hemiparesis	1

M, male; F, female; Fop, frontal operculum; FPop, frontoparietal operculum; TPop, temporoparietal operculum; L, left; R, right; FCD, focal cortical dysplasia; N, normal; NA, not applicable.

The study was approved by the University of Montreal Hospital Center ethics board and conformed to The Code of Ethics of the World Medical Association (Declaration of Helsinki). Informed consent was obtained from all patients.

### 2.2 MRI acquisition

For each patient, a high-resolution structural MRI was performed at three timepoints for a total of 30 scans: within a year prior to surgery (t_0_) and at 6 months (t_1_) and 12 months postoperatively (t_2_). T1w images (TR = 8.1 ms; TE = 3.8 ms; flip angle = 8°; voxel size = 1 × 1 × 1 mm; FOV = 230 × 230 mm) and diffusion-weighted imaging (DWI) sequences at a high angular resolution (60 noncollinear diffusion directions, *b* = 1,500 s/mm^2^, and one *b* = 0 s/mm^2^ image) were acquired on a 3 T Achieva X MRI (Philips, the Netherlands).

### 2.3 Image processing and construction of connectivity matrices

We used Tractoflow version 2.3.0 (51), a novel fully automated tractography pipeline, to process all preoperative (t_0_), 6-month postoperative (t_1_) and 12-month postoperative (t_2_) raw T1 and diffusion-weighted images. Rather than using the default tractogram generated from Tractoflow, intermediate outputs were processed with a cutting-edge surface-informed anatomically-constrained probabilistic tracking algorithm computed from fiber orientation distribution functions (fODFs), namely SET version 1.1 (41). The resulting whole-brain tractograms have been shown to be more anatomically plausible and robust to the gyral bias of conventional tractography techniques (41). More details regarding the method we employed to generate tractograms and the benefits of SET are available in Obaid et al. (3).

The Freesurfer-generated surface (52) of preoperative (t_0_) native T1w images was used to segment the cortex and the subcortical gray matter into 249 parcels [246 Brainnetome regions (53), brainstem -247-, left cerebellum -248- and right cerebellum -249-]. Since Freesurfer erroneously creates an artifactual surface at the edge of surgical cavities—resulting in misplaced labels in the white matter—we opted to compute the postoperative parcellations using preoperative data. In this regard, preoperative parcellations were registered to native T1w images at t_1_ and t_2_ using ANTs diffeomorphic registration (54, 55).

**Figure 1:**
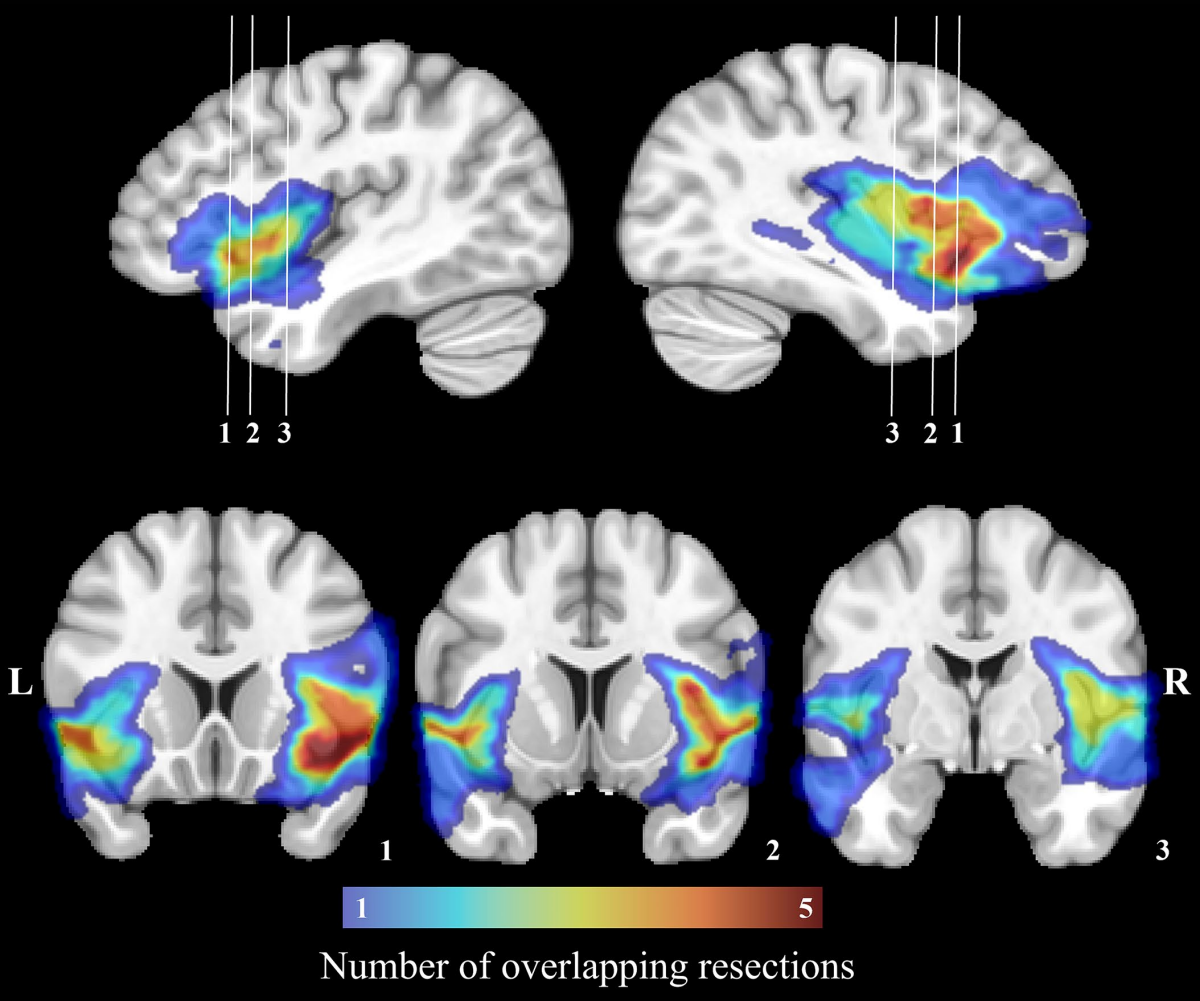
Manually drawn surgical cavities registered to the MNI-152 space. The color bar corresponds to the number of overlapping resections at each voxel. The region with the most overlapping resections is located on the right insula.

For every patient, a mask of the resection cavity was manually drawn on native T1w images at both postoperative timepoints (t_1_ and t_2_) using MI-Brain visualization tool (56). To account for the progressive remodeling of the shape and size of the resection cavity over time, both masks were nonlinearly registered to the MNI-152 space and then merged. The merged resection mask pertaining to a patient was then independently registered to its native t_0_, t_1_ and t_2_ T1w images and the portions of the labels overlapping with the registered merged mask were excluded. Hence, despite being in distinct native spaces, the included labels at all three timepoints were identical within a subject (subject-wise timepoint-invariant parcellation). On the other hand, because each patient underwent a tailored surgical resection, the resulting masks and included labels differed between subjects. Rather than creating a group mask, we treated every resection mask independently to allow analysis of the potentially plastic peri-resection areas (38, 57) in patients who underwent smaller corticectomies (these areas would have been discarded in a group resection mask). Figure 1 illustrates the overlap of all merged resection cavities in the MNI-152 space.

**Figure 2:**
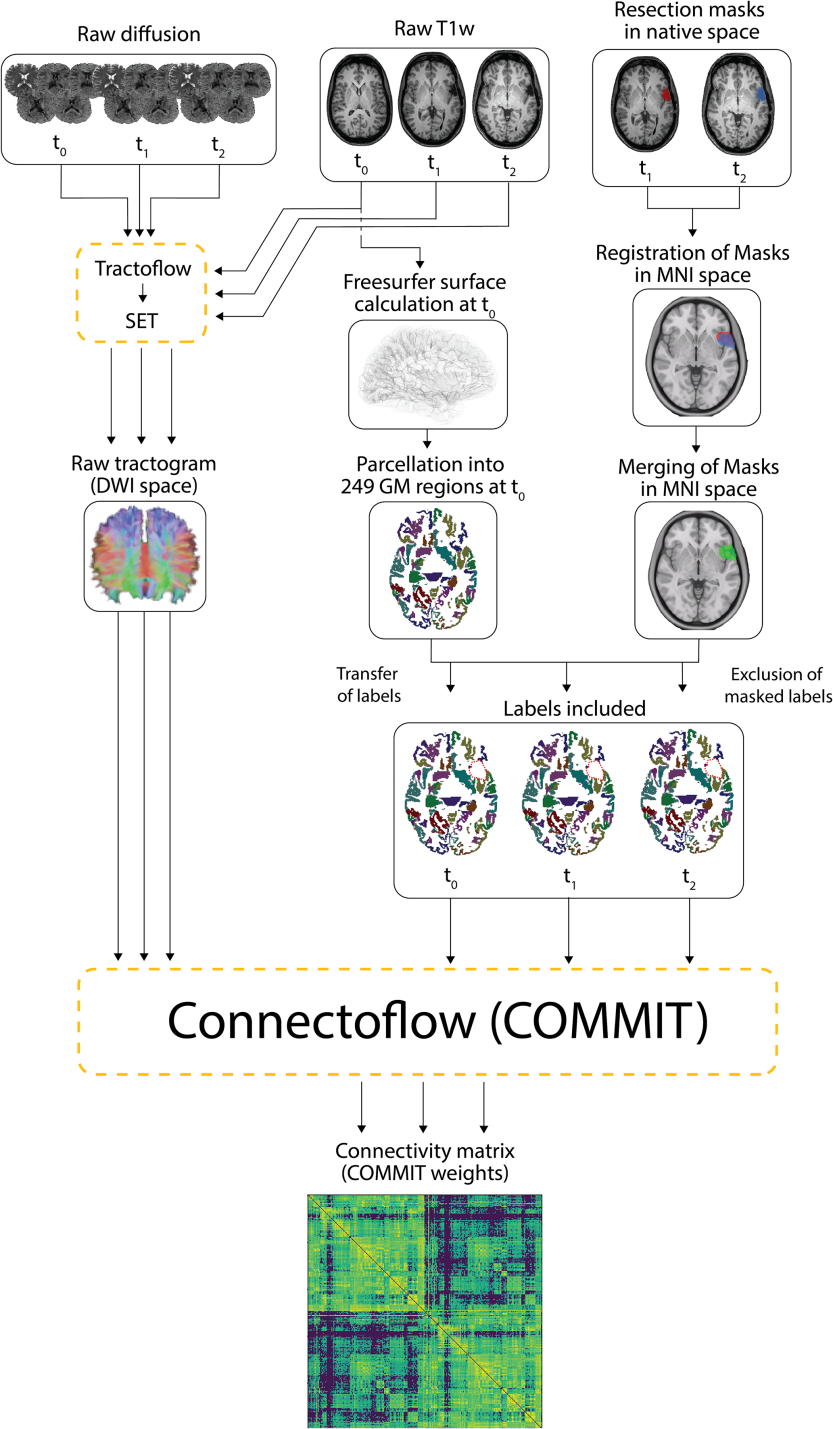
Processing pipeline. Raw T1 and diffusion-weighted images were processed using Tractoflow and SET to generate raw tractograms. The Freesurfer surfaces calculated on native T1w images at t_0_ were segmented into 249 cortical/subcortical regions. Resection masks delineated on native T1w images at t_1_ and t_2_ were registered in the MNI space and merged. The 249 labelled regions computed at t_0_ were individually registered to t_1_ and t_2_ native T1w images while the merged masks in MNI space were registered to t_0_, t_1_ and t_2_ native T1w images (*transfer of labels*). The labels and merged masks registered to the same T1 space were combined to exclude portions of labels falling within the resection cavity (*exclusion of masked labels*) and retain unresected and hence included labels. The included labels and the raw tractograms were used by Connectoflow to derive COMMIT-weighted structural connectivity matrices. GM, gray matter.

The tractograms built with SET and the mask-filtered labels were combined and processed in Connectoflow version 1.1.1 (58), a comprehensive structural connectivity pipeline. As part of Connectoflow processing, COMMIT was used to filter the SET-derived tractogram and generate the COMMIT weight (CW) of individual streamlines. In short, COMMIT allowed to compute the signal contribution of each streamline to the raw dMRI images (46, 47). The COMMIT-derived contribution of each streamline was then multiplied by its length and divided by the average length of the bundle in which the streamline travels to generate the CW of individual streamlines (48). The CWs of all streamlines connecting two cortical/subcortical labels were then summed in Connectoflow to compute the CW of each white matter connection, the metric of CS in our study. Compared to the commonly used streamline count, the CW of a connection is a more accurate and more representative estimate of CS that better reflects the underlying microarchitecture of fibers (46–48). The final step of Connectoflow was the creation of structural connectivity matrices from CW values. Figure 2 summarizes the processing flowchart used to generate the structural connectivity matrices.

To improve statistical power, matrices of patients exhibiting a left-sided epileptic focus (four patients) were sided-flipped. A threshold based on preoperative data was applied such that connections with a CW of zero in 10% or more of patients at t_0_ were removed from the t_0_, t_1_ and t_2_ matrices. This filtering step allowed us to study anatomically plausible connections by removing spurious connections and inappropriately under-reconstructed bundles in difficult-to-track regions.

### 2.4 Longitudinal analysis of connectivity strength and graph theoretic measures

We computed 249 × 249 whole-brain COMMIT-weighted matrices for all patients at all three timepoints. We also built submatrices connecting (i) the 124 ipsilateral regions (ipsilateral hemisphere subnetwork), (ii) the 124 contralateral regions (contralateral hemisphere subnetwork) and (iii) the six contralateral insular subregions (contralateral insular subnetwork). In addition to edge-based comparisons of CS, graph theory analyses were performed to characterize the changes in network topological properties following surgery (59). Comparisons of CS were carried out on whole-brain matrices while analyses of graph theoretic measures were performed both on whole-brain networks and on subnetworks. Graph theory analyses of specific subnetworks were performed to evaluate the independent reorganization of different brain areas. This approach has been previously shown to provide valuable information regarding the architecture of regional networks in focal epilepsy (30).

Using scripts from the Brain Connectivity Toolbox (60, 61), we computed undirected COMMIT-weighted adjacency matrices and derived various regional (betweenness centrality, clustering coefficient, local efficiency, and nodal strength) and global graph theoretic measures (characteristic path length, global efficiency, small-worldness, average betweenness centrality, average clustering coefficient, and average nodal strength). The definitions of the graph theoretic metrics are summarized in Supplementary Table S1. To assess postoperative changes in connectivity over time, we performed longitudinal analyses by comparing matrices of CS and graph theoretic measures between (a) t_0_ and t_1_ (early postoperative changes) and (b) t_1_ and t_2_ timepoints (late postoperative changes). To simplify interpretation, these results are indexed to the earlier timepoint in the comparison (i.e., a reported increase in a metric indicates that the later timepoint was increased as compared to the earlier timepoint). Moreover, to assess when most of the postoperative structural remodeling occurred, we compared the magnitudes of the absolute differences in CS between the early (t_0_-t_1_) and late (t_1_-t_2_) postoperative intervals. The absolute differences for both time intervals were calculated using the following formulas:


$$\begin{array}{l} \;\mathrm{Absolute}\;\mathrm{Differences}\;for\;\mathrm{the}\;\mathrm{early}\;\mathrm{postoperative}\;\mathrm{interval}=\\ \left|CWt1_{ij}-CWt0_{ij}\right| \end{array}$$

$$\begin{array}{l} \mathrm{Absolute}\;\mathrm{Differences}\;for\;\mathrm{the}\;\mathrm{late}\;\mathrm{postoperative}\;\mathrm{interval}=\\ \left|CWt2_{ij}-CWt1_{ij}\right| \end{array}$$

where CW represents the COMMIT weight at times t_0_, t_1_, and t_2_, averaged across all participants for a given connection defined by the labels *i* and *j*. Given that the matrix of all connections was symmetrical, the condition *i* < *j* was imposed, such that only the lower triangular matrix, excluding the main diagonal, was considered. All hypothesis tests were performed using paired sample *t*-tests with permutation testing, using 1,000 iterations. Paired sample *t*-tests compare pairs of observations from the same participants (o_1_ and o_2_). For each iteration, a random number of subjects had their o_1_ and o_2_ values switched, and the paired sample *t*-test was repeated. The original *t* statistic stemming from the non-permuted data was then compared against the null distribution of *t* statistics created from all permutations to obtain the *p* value. This non-parametric approach was selected to avoid issues with non-normality of the data. For CS, CS absolute difference, and regional graph theory comparisons, thresholds of *p* ≤ 0.001 and *q* ≤ 0.05 were used for uncorrected and false discovery rate (FDR)-corrected analyses, respectively. For comparisons of global measures, an uncorrected threshold of *p* ≤ 0.05 was applied.

### 2.5 Visualization

Three dimensional projections of structural connections and regional nodes were illustrated using Visualization Toolkit version 9.1 (62) for analyses of COMMIT-weighted matrices and graph theoretic measures. The ipsilateral side of the 3D reconstructed brain corresponds to the side of seizure onset/surgical resection.

## 3 Results

### 3.1 Longitudinal changes in connectivity strength

**Figure 3:**
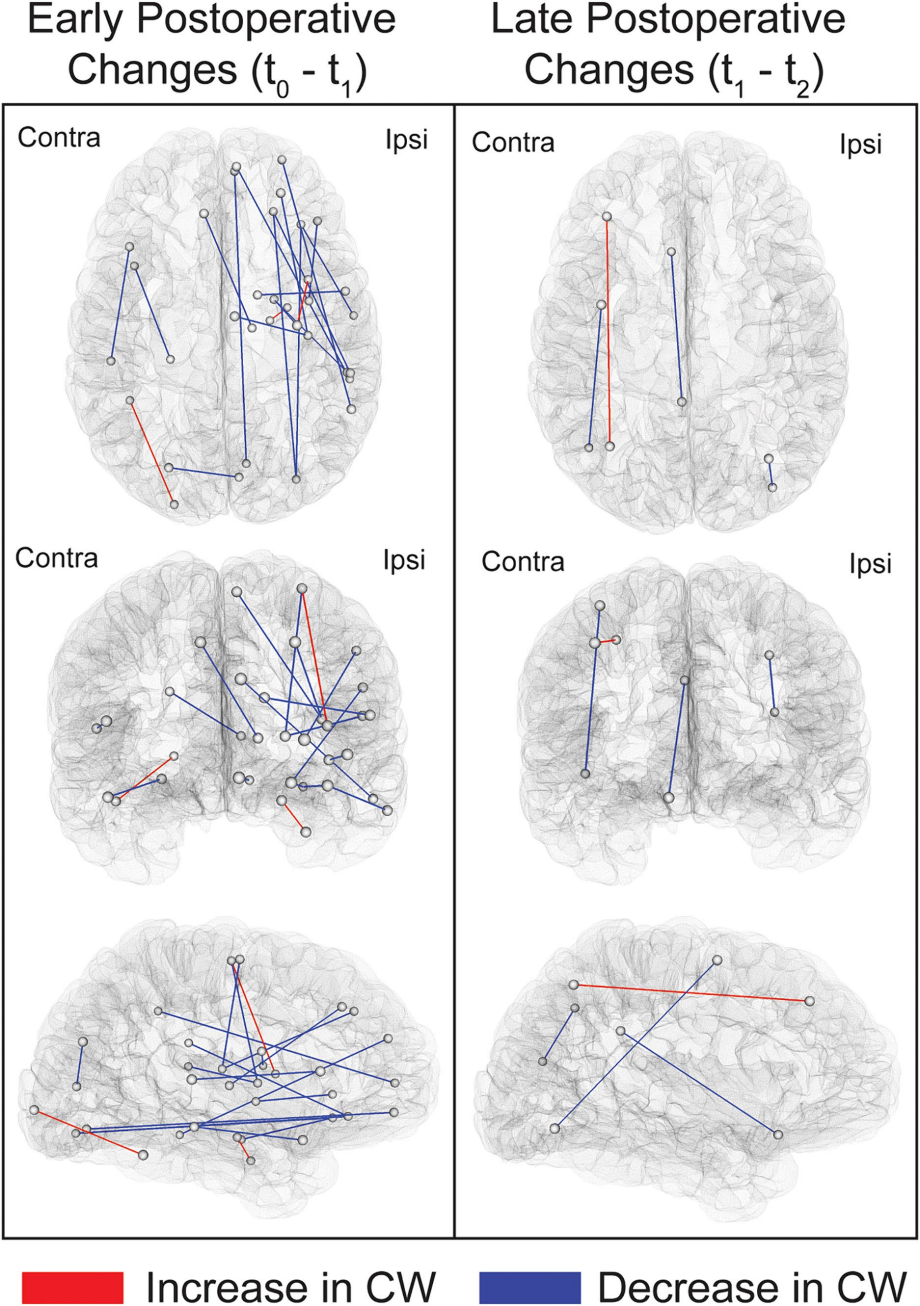
Illustration of the links showing postoperative changes in COMMIT weights. Connections exhibiting changes between t_0_ and t_1_ (early postoperative changes) and between t_1_ and t_2_ (late postoperative changes) are depicted. Comparisons were performed using paired sample *t*-tests at a threshold of *p* < 0.001 uncorrected. The connections shown in this figure are listed in Supplementary Table S2. Ipsi, ipsilateral; Contra, contralateral.

Comparing t_0_ to t_1_ revealed widespread, primarily ipsilateral, early decreases in CW following surgery. The pattern of increases in CW was, in contrast, limited to three bundles (Figure 3; Supplementary Table S2). Notably, two of these three connections were located in regions adjacent to the resection cavity, namely the link between the dorsal insula and the superior portion of the precentral gyrus and the link between the hippocampus and parahippocampal gyrus. The third connection revealing an early increase in CW was the one adjoining the fusiform gyrus to the occipital pole on the contralateral side. Moreover, comparing t_1_ to t_2_ revealed a more restricted distribution of late structural connectivity changes (Supplementary Table S2). None of these comparisons survived FDR correction, and therefore the results reported were obtained using an uncorrected threshold of *p* ≤ 0.001. The average COMMIT-weighted connectivity matrices at t_0_, t_1_ and t_2_ are illustrated in Supplementary Figures S1–S3.

**Figure 4:**
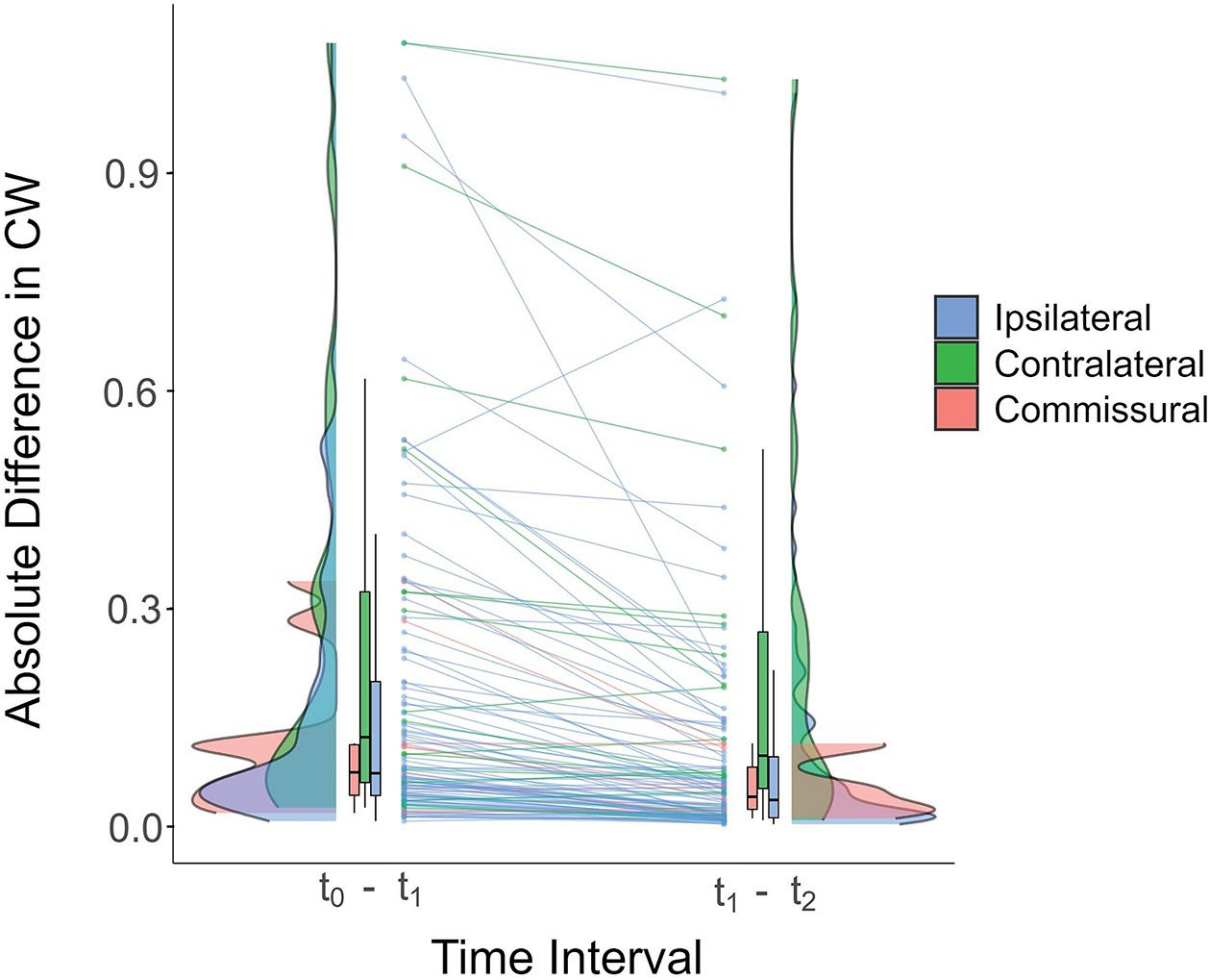
Raincloud plot of the absolute differences in COMMIT weights for ipsilateral (blue), contralateral (green) and commissural (pink) connections in the early (t_0_-t_1_) and late (t_1_-t_2_) postoperative intervals (averaged across all participants). Each line connects the averaged differences of the two time intervals for the same connection. For most connections, the magnitude of the difference was greater in the t_0_-t_1_ interval, suggesting that changes in connectivity strength tend to occur early following surgery. Connections exhibiting a statistically significant between-interval difference in absolute differences of COMMIT weights are shown (for illustrative purposes, an uncorrected threshold of *p* ≤ 0.005 was used). Horizontal black lines in the box plots correspond to the medians, the edges of the boxes illustrate the 75th and 25th percentiles, and vertical lines depict ranges, excluding outliers. The distribution densities of the absolute differences for the connections included in the plot are shown beside the boxes. CW, COMMIT weight.

Since most of the changes were in the early postoperative phase, we also sought to assess whether the magnitudes of absolute differences in CW were greater for the t_0_-t_1_ than for the t_1_-t_2_ interval. Out of the 14 connections for which the absolute differences between those two time intervals differed (*p* ≤ 0.001 uncorrected), 13 (93%) revealed a greater change in the t_0_-t_1_ interval. Despite the lack of survival following FDR correction, this analysis suggests that most changes occur in the first six months following surgery. Figure 4 shows connections with differences that are significant under a more permissive, uncorrected threshold of *p* ≤ 0.005, which was chosen in order to include more connections and therefore better illustrate the overall trend.

To determine if the pattern of increased connectivity was preferentially lateralized, chi-square tests were used to evaluate the early postoperative changes in CS in ipsilateral and contralateral connections. Interestingly, 49% of connections in the contralateral hemisphere exhibited a postoperative increase in CW compared to 38 % on the ipsilateral side (chi-square test of independence; χ^2^ = 80.59; *p* < 2.2e-^16^). Moreover, out of all the intrahemispheric connections that displayed an increase in CW, 56% were located on the contralateral side (chi-square goodness of fit test; χ^2^ = 45.17; *p* < 1.8e-^11^).

### 3.2 Longitudinal changes in network topology

**Figure 5:**
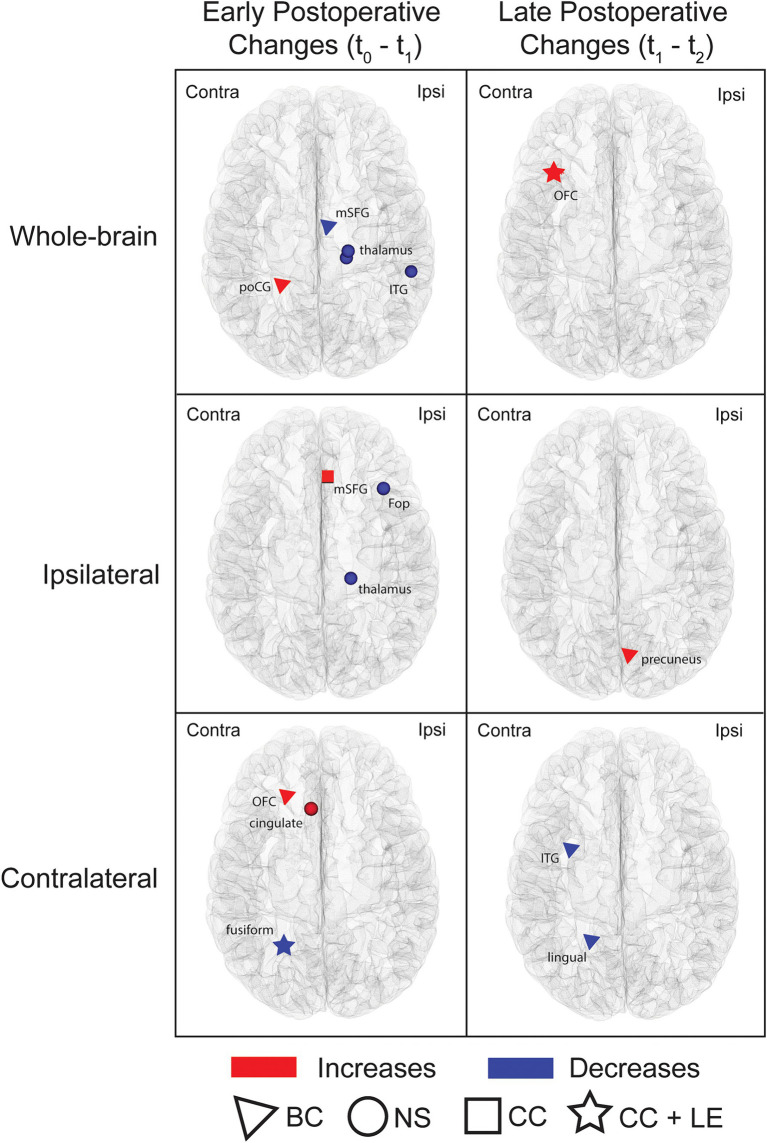
Nodes showing postoperative changes in regional graph theoretic measures. Whole-brain networks and subnetworks were analyzed. Nodes exhibiting changes between t_0_ and t_1_ (early postoperative changes) and between t_1_ and t_2_ (late postoperative changes) are shown. The symbols highlight areas that exhibited changes in graph theoretic metrics (triangle = betweenness centrality; square = clustering coefficient; circle = nodal strength; star = regions with changes in both clustering coefficient and local efficiency). Comparisons were performed using paired sample *t*-tests at a threshold of *p* ≤ 0.001 uncorrected. The nodes highlighted in this figure are detailed in Supplementary Table S3. Ipsi, ipsilateral; Contra, Contralateral; BC, betweenness centrality; NS, nodal strength; CC, clustering coefficient; LE, local efficiency; poCG, postcentral gyrus; mSFG, medial superior frontal gyrus; ITG, inferior temporal gyrus; OFC, orbitofrontal cortex; Fop, frontal operculum.

Graph-theory analyses of whole-brain networks and subnetworks identified early and late postoperative changes in various regional measures (uncorrected threshold of *p* ≤ 0.001; Figure 5; Supplementary Table S3). When comparing t_0_ to t_1_, differences were observed for the ipsilateral, contralateral and whole-brain networks. On whole-brain analyses, the ipsilateral medial frontal area showed a decrease in betweenness centrality whereas the contralateral postcentral gyrus showed an increase; within the same network, the ipsilateral inferior temporal area and posterior thalamus exhibited a decrease in nodal strength. Within the ipsilateral hemisphere subnetwork, an increase in clustering coefficient was found in the medial frontal area and a reduction in nodal strength was observed in the frontal opercular region and posterior thalamus. Finally, analysis of the contralateral hemisphere subnetwork revealed increased betweenness centrality and nodal strength in the orbitofrontal and anterior cingulate areas, respectively, and reduced clustering coefficient and local efficiency in the mesiotemporal region.

Contrasting t_1_ and t_2_ networks also showed changes. Comparisons of whole-brain networks revealed an increase in clustering coefficient and local efficiency in the contralateral orbitofrontal area while analyses of subnetworks revealed higher betweenness centrality in the ipsilateral medial parietal lobe (ipsilateral hemisphere subnetwork) and lower betweenness centrality in the contralateral inferior temporal and medial occipital areas (contralateral hemisphere subnetwork).

None of the early or late postoperative changes survived FDR correction for multiple comparisons, and analysis of the contralateral insular subnetwork revealed no statistically significant changes.

We also found differences in global measures (uncorrected threshold of *p* ≤ 0.05). Interestingly, all the changes were observed early following surgery (t_0_ vs. t_1_). At 6 months, the characteristic path length was higher while the small-worldness and global efficiency were lower in both whole-brain network and ipsilateral subnetwork analyses. There were no changes in average betweenness centrality, average clustering coefficient or average nodal strength.

## 4 Discussion

Over the past fifteen years, longitudinal dMRI studies have elucidated the white matter remodeling underlying functional recovery following surgery for various types of focal epilepsy (34–38, 63–65). Cumulative work in temporal lobe epilepsy (TLE) has shown that improvements of cognitive functions after temporal lobe resections are underpinned by manifest morphological white matter plasticity (37, 38). These findings in extra-insular epilepsy, combined with the notable recovery of postoperative neurological deficits frequently seen following OIE surgery (15, 22), provide a reason to expect that comparable adaptive changes may develop after operculo-insular resections for focal epilepsy. In this study, we implemented a comprehensive pipeline that incorporates novel tractography tools and advanced connectome-building approaches to assess the longitudinal pattern of structural connectivity changes in patients undergoing resective surgery for OIE. Towards this goal, tractography-derived structural connectomes of patients with long-standing OIE were computed before surgery and at two postoperative timepoints. We observed a widespread bilateral pattern of bidirectional changes, most of which were reductions in connectivity and involved the ipsilateral hemisphere. Moreover, the majority of increases in connectivity were in the contralateral hemisphere and in regions near the resection cavity. Finally, most of the changes, whether increases or decreases, occurred in the first six months following surgery.

### 4.1 Postoperative reductions in connectivity

Analyses of whole-brain networks following surgery showed an extensive pattern of reductions in CS involving frontal, parietal, temporal, occipital and insular areas, the thalamus, the globus pallidus and the caudate nucleus. Most of these variations occurred early following surgery and involved the surgical hemisphere. Moreover, regional graph theory analysis revealed concordant postoperative alterations in network topology. We found predominantly ipsilateral multifocal decreases in local connectivity-reflecting graph theoretic measures, most of which appeared in the first six months following surgery. Interestingly, the preferentially ipsilateral pattern of reduced postoperative connectivity highlighted by our analyses seems to reflect the longitudinal changes in the white matter microstructure (35–38, 63) and the progressive reorganization of structural networks (35, 40) seen following surgery for TLE (35–38, 40, 63, 65). Indeed, although the quantitative measures of postoperative changes varied between studies, with some using fractional anisotropy (FA) or related metrics (35–38, 63, 65) and others exploiting the inter-regional streamline count (40), a common pattern of predominantly ipsilateral reductions in FA, CS or nodal strength emerges.

While the exact pathophysiology of such white matter alterations in the surgical hemisphere remains uncertain, studies in TLE have shown that many of the altered tracts tend to be in the proximity of or connected to the resected area (35, 36, 38, 63, 65), suggesting that Wallerian degeneration might play a significant role (35, 36, 38, 63, 65). In our study, the same surgical mask was applied to postoperative and preoperative images, and tracts connected to the masked cortical labels were excluded from the analysis at all three timepoints. It is therefore unlikely that the observed ipsilateral connectivity reductions were due to distal degeneration of connections severed during surgery. On the other hand, previous work has revealed that Wallerian degeneration can occur along bundles indirectly connected to the resected area (65), raising the possibility that this second-degree process may in part explain our findings. Another possible explanation may come from the potential for white matter abnormalities to revert following postoperative improvement in seizure control (36). There is cumulative evidence in focal epilepsy that repetitive seizures may be associated with adaptive axonal sprouting and neurogenesis, ultimately leading to white matter alterations within the epileptic network (3, 28, 30, 66). In this regard, our group has recently revealed a pattern of “hyperconnected” bundles within the epileptic network of OIE (3). Interestingly, the distribution of “hyperconnections” observed in that previous study was not restricted to insular tracts but rather involved a pattern that, just like the distribution of postoperative reductions observed in the current investigation, was extensive and primarily ipsilateral (3). Based on these findings, it is conceivable that the decrease in connectivity observed in our population of patients—all of whom had a favorable postoperative seizure control—may be related to the reversal of pathologically “hyperconnected” regions within the ipsilateral OIE network. This idea of postoperative structural normalization is further supported by recent reports revealing an association between a reduction in CS, nodal strength or quantitative anisotropy (a measure correlated with FA) in ipsilateral tracts and seizure freedom following surgery for TLE (35, 40).

### 4.2 Postoperative increases in connectivity

A more restricted pattern of postoperative increases in CS and nodal graph theoretic measures was observed. Most of these changes appeared early following surgery and, in contrast to the pattern of decreased connectivity, on the contralateral side. This propensity for the contralateral side to show strengthening of structural connections was further emphasized by our analysis of the lateralized distribution of connectivity changes. The analysis revealed that, as compared to the ipsilateral side, the unaffected healthy hemisphere was more likely to show increases in CS (49% of contralateral changes compared to 38% of ipsilateral changes) and contained the majority of such changes (56% of all increases). Moreover, the results of global graph theory analyses showed an early decrease in global efficiency (increase in characteristic path length) and small-worldness within ipsilateral and whole-brain networks. Global efficiency is a measure of integration that reflects the efficacy of information flow in a circuit, while the small-worldness describes the network’s ability to balance both segregation and integration (29, 67–69). The pattern of decreases in global efficiency and small-worldness affecting exclusively whole-brain and ipsilateral networks may therefore indicate an overall less integrative network driven by ipsilateral but not contralateral alterations, changes that are consistent with the predominantly ipsilateral widespread pattern of decreased connectivity. These findings, combined with the lack of decreases in average clustering coefficient on whole-brain analyses and the preferentially contralateral distribution of connectivity increases, suggest preserved segregation conferred by structural remodeling of the nonsurgical hemisphere. In this sense, previous studies on temporal (37) and extratemporal epilepsies (34) have shown postoperative increases of structural CS (34) and FA (37) in contralateral bundles, a subset of which were correlated with improvements in cognitive function following surgery (34, 37). Along the same lines, albeit focusing on a different etiology, reports on traumatic brain injury support the role of the contralateral hemisphere in functional recovery. In a recent study by Jang et al. (70), motor recovery at 3 months in a patient with a left traumatic supplementary motor area (SMA) injury was associated with a compensatory volume increase in the right tractography-reconstructed SMA-corticofugal tract (70). This suggests that recovery from OIE surgery may share contralateral mechanisms akin to those observed in the SMA syndrome, a well-documented condition known for its hallmark feature of complete or near-complete functional recovery within weeks (71). While these adaptive structural changes in the uninjured hemisphere are also supported by functional MRI and neuropsychological investigations (34, 72, 73), other reports stand in contrast by unveiling a more important role of white matter tracts on the side of resection (38). In this regard, many studies on focal epilepsy revealed primarily ipsilateral postoperative increases in FA (36, 38, 63), some of which were related to functional improvement (38). Moreover, large analyses of patients with ischemic lesions also support the idea that remodeling in ipsilateral networks is more important than in contralateral networks and further highlight that most plasticity occurs in connections located near the injury (57). In the current investigation, an increase in CS was also observed in two ipsilateral bundles (dorsal insula-precentral gyrus and rostral hippocampus-parahippocampal gyrus) and, remarkably, both connections were located in the vicinity of the resected operculo-insular area. Taken together, these results imply that following surgery for OIE, increases in connectivity may develop predominantly in regions located close to the surgical cavity or on the contralateral side.

The exact mechanism underlying the increase in CS following surgery for focal epilepsy is incompletely understood. However, there is reason to suspect that the observed rearrangements constitute functionally adaptive responses (34, 36–38, 63). Jeong et al. (34) proposed that the contralateral increase in CS following resective surgery for focal epilepsy reflects an underlying increase in axonal density and that such remodeling may mediate the release of a reserve, previously suppressed by ongoing seizures, that becomes capable of overcoming surgically induced memory impairments. Along the same lines, analyses of white matter microstructure in TLE have suggested that improvements in FA following surgery may be due to behaviorally adaptive increases in myelination, fiber density or axonal regeneration (35, 36). With respect to OIE surgery, there is evidence from multicentric data that despite a significant proportion of patients developing postoperative deficits (primarily in motor, sensory and language functions), most recover fully and rapidly (10, 12, 15, 22). For instance, our group has demonstrated in a recent meta-analysis that while 42% of patients experience neurological impairments following resective surgery for OIE, 78% of deficits are transient, of which 69% resolve completely within three months (15). This unusual pattern of recovery was also found in the current study—all five patients who developed postoperative deficits fully recovered within six months. Interestingly, this short delay to recovery is remarkably consistent with the longitudinal analysis of absolute differences in CW which revealed a strong predilection for early (93%) rather than late (7%) changes. Based on these findings, it is reasonable to believe that the observed pattern of increased structural connectivity may constitute a responsive mechanism that arose to supplant the functions of the injured operculo-insular area (34–36). Yet, one could argue that these changes partly reflect a mechanism that develops to take over the functions of peri-insular structures rather than the insular cortex. While resection of the insular cortex itself may result in motor, sensory and language deficits, there is growing evidence that these impairments following OIE surgery may instead be caused by extra-insular lesions (10, 15, 74, 75). Motor impairments may be attributable to corona radiata strokes resulting from iatrogenic injury to long insular arteries arising from the second segment of the middle cerebral artery (10, 15, 74). Sensory and language deficits may stem from transgression of the parietal and dominant frontal (pars opercularis and/or triangularis) opercula, respectively, during transopercular insular resections (10, 15, 74, 75). Although the biological substrate of the adaptive process in OIE remains uncertain, it may involve an increase in axonal sprouting, myelination or neurogenesis-related fiber density (34–36).

### 4.3 Study design and methodological considerations

The current study was designed to provide valuable and trustworthy data regarding the structural remodeling following surgery for OIE. We recruited 10 patients with OIE, all of whom were longitudinally analyzed at three predefined timepoints. We gathered data at two postoperative timepoints in order to assess the gradual progression of structural changes. While many reports of focal epilepsy have used a single postoperative timepoint (34, 38, 40, 64, 76, 77), some studies have instead collected imaging data at multiple timepoints (36, 39). These studies have highlighted the benefits of longitudinal analyses in the assessment of progressive plasticity following epilepsy surgery, prompting us to use a similar approach to evaluate changes after OIE surgery. With respect to the imaging timescale, the delays between surgery and postoperative scans were chosen to allow enough time to capture the majority of structural remodeling. In this regard, previous series on TLE have shown that most white matter plasticity occurs in the first three to six months following surgery and that subsequent remodeling is often negligible or limited (35, 36, 65). In a study by Liu et al. (39), forniceal changes were seen as early as the first few days following surgery. In another study by Winston et al. (36), remodeling occurred in the first three to four months following temporal lobectomy while plasticity beyond that timeframe was trivial. For those reasons, it could be argued that assessing changes at earlier timepoints (i.e., multiple timepoints in the first six postoperative months) may be more relevant to characterize the remodeling following OIE surgery than evaluating late plasticity (i.e., between six and 12 months). Yet, the objective of our study was to examine the structural changes in a time range that matches the pattern of functional improvement seen after OIE surgery. Since most patients recover within the first six postoperative months, with only a minority showing later improvement (15), we opted to gather imaging data at six and 12 months.

From a methodological standpoint, we opted to use specific approaches and tools that reliably evaluate structural networks and address challenges associated with the presence of a surgical lesion and the longitudinal aspect of our analysis. We used an elaborate pipeline that integrates state-of-the-art quantitative structural connectivity methods to generate anatomically plausible connectomes and relevant measures of CS. The use of SET and its built-in fODF-based anatomically-constrained probabilistic tracking algorithm favored the reconstruction of fiber intersections (42), curvatures (43) and juxtacortical fanning (41), resulting in coherent streamlines that are compelled to terminate in the gray matter and cover the cortex more homogeneously. As compared to standard tracking algorithms, SET provides a more accurate representation of streamlines and enables the generation of denser and more representative connectomes. We also used COMMIT to filter the SET-derived tractograms and compute the CWs (46, 47). Briefly, COMMIT assumes that the microstructural fiber properties are constant along the length of a tract and, by comparing the reconstructed streamlines to the initial diffusion signal, decomposes the intrinsic signal contribution of each streamline in the tractogram (46–48). By doing so, one normalized value (weight) is estimated per streamline (rather than one value per voxel) (46–48) and the individual weights of all streamlines connecting two gray matter parcels can be summed to obtain the CW of a connection. In contrast to the traditional streamline count, the CW is a more precise and informative quantitative metric of CS (48, 50). The CW is not only less influenced by variations in the morphology of bundles and by the selection of tracking criteria (43, 50, 78), but also better reflects the underlying axonal microarchitecture (46, 47). Moreover, we removed aberrant connections from connectivity matrices using a two-step method. We first exploited COMMIT to exclude inappropriately reconstructed streamlines (46, 47). We then thresholded matrices such that a connection was only retained and analyzed at all three timepoints if its CW before surgery was above zero across 90% of participants (79, 80), thereby discarding erroneously unreconstructed bundles and spurious tracts. This sequential approach was used to limit the number of false positive and false negative connections, and therefore favored the selection of reliable bundles and the computation of valid graph theoretic measures (81). Finally, we applied a manually delineated resection mask at all three timepoints. Instead of considering only one postoperative image, masks were fused across both postoperative timepoints, allowing to account for the gradual remodeling of the surgical cavity. We also treated every mask independently (each patient had a unique mask) rather than using a group mask created from the union of all resections (35, 36, 40). Group masks have been used in previous studies of TLE and have the benefit of simplifying longitudinal comparisons of structural networks (35, 36, 40). When studying standardized surgeries such as the temporal lobectomy, the use of group masks may be justified because they deviate minimally from individual masks. Our investigation focused on surgery for OIE, a rather heterogeneous procedure for which the exact location and the extent of resections may vary between patients (10–12, 15, 22). Considering every mask separately allowed the analysis of all non-resected areas. These included regions with great potential for plasticity such as the peri-resection zones (38), some of which may have been inappropriately hidden by group masks in patients undergoing smaller resections.

While our study has uncovered informative results, it has some limitations that should be mentioned. First, our sample size was relatively small. To maximize the number of participants, we combined patients regardless of the underlying etiology of OIE, the specific location of the operculo-insular seizure onset, and the subregions that were resected. In accordance with similar studies in TLE (40, 76, 77), we side-flipped patients with a left-sided resection. This step allowed us to assess the whole cohort uniformly. While we recognize that evaluating subgroups separately would have avoided potential biases related to tracking and could have revealed differential distributions of postoperative structural changes, the rarity of OIE surgeries necessitated the use of an inclusive approach. Even so, the fact that all 10 patients underwent three scans (for a total of 30 observations) compares favorably to the sample size of longitudinal white matter analyses in surgery for TLE (64, 65). Second, the limited number of postoperative impairments (five cases) and the heterogeneity of these deficits prevented the assessment of the relationship between structural remodeling and functional improvement. It would also have been interesting to compare patients who developed a deficit that recovered to the ones who did not exhibit functional improvement. Such analysis was however precluded by the fact that all patients included in our study entirely regained their functions. Third, we did not evaluate neuropsychological performances, which are known to be affected following surgery for OIE (82). Fourth, all patients underwent an open resection; therefore, the observed postoperative connectivity changes cannot be generalized to minimally invasive ablative approaches such as radiofrequency ablation or laser interstitial thermal therapy. Because these stereotactic approaches target smaller volumes (83, 84), they may be better at preserving eloquent regions and avoiding neurological deficits (15). Thus, a more restricted pattern of compensatory connectivity changes might be expected in these cases. Fifth, despite our efforts in optimizing the registration of resection masks and gray matter labels, misalignments may have occurred. In particular, we opted to calculate postoperative parcellations using preoperative data. This approach allowed to avoid the erroneous computation of labels within the resection cavity that typically occurs when Freesurfer processing and Brainnetome parcellation are performed on postoperative images. However, anatomical distortion following surgery may have led to inaccurate registration and mislabelling, thereby resulting in comparisons of non-corresponding regions. Sixth, despite providing clear advantages, SET and COMMIT are limited by surface reconstruction and dMRI image quality, respectively (41, 46). In addition, SET can only reconstruct a single fanning distribution in the juxtacortical region (i.e., it cannot account for crossing fibers immediately adjacent to the cortex) (41), and COMMIT assumes that the microstructural properties remain constant along white matter trajectories, potentially reducing the CS of longer streamlines (46). Finally, none of our results survived FDR correction. In this regard, the numerous nodes and edges of connectivity analyses increase the number of comparisons, which can result in a prohibitive corrected threshold and a high rate of type II errors (29, 34). The high-resolution parcellation used in our study favored a more detailed assessment of structural variations, but also led to a highly restrictive corrected threshold that likely masked some changes. Moreover, the low statistical power of our analysis likely contributed to the lack of survival of small but informative differences following FDR correction. Those reasons, combined with our intention to be sensitive to subtle rearrangements, justified the use of an uncorrected but strict threshold (*p* ≤ 0.001) to report our findings (37). While we recognize that reporting uncorrected data may increase the risk of false positive results, our assessment of structural connectivity changes following OIE surgery is unprecedented and should therefore be considered exploratory. Hence, the use of a less restrictive threshold was deemed appropriate. An alternative approach could have involved a corrected comparison with a larger effect size. For instance, comparing connectivity matrices between t_0_ and t_2_ may have increased the power of the analysis and disclosed regions that survived FDR correction. However, in order to specifically assess whether the timing of structural remodeling matched the pattern of functional recovery, we instead focused on contrasting the phase exhibiting most of the clinical recovery (first six months) to the phase displaying negligible improvement (between six and 12 months). This longitudinal analysis allowed to highlight a differential pattern of early and late plasticity that corresponds to the postoperative course of functional recovery.

We unveiled, for the first time, the longitudinal changes in structural connectivity following surgery for OIE. Using an elaborate pipeline that incorporates reliable tracking algorithms and quantitative connectomic tools, we revealed a widespread bilateral pattern of postoperative changes. The pattern included extensive, primarily ipsilateral reductions in connectivity along with connectivity increases distributed predominantly around the resection cavity and in the contralateral healthy hemisphere. Interestingly, most structural changes occurred within the first six postoperative months, a timeframe that is consistent with the rapid improvement of deficits engendered by operculo-insular resections for focal epilepsy. These preliminary findings provide unique information regarding the structural plasticity following OIE surgery that may contribute to our understanding of its peculiar course of recovery. Future studies with a larger sample size are needed to further characterize these morphological changes and validate if they are in fact related to the atypical functional recovery associated with surgery for OIE.

## Data availability statement

The raw data supporting the conclusions of this article will be made available by the authors, without undue reservation.

## Ethics statement

The studies involving humans were approved by University of Montreal Hospital Center ethics board. The studies were conducted in accordance with the local legislation and institutional requirements. The participants provided their written informed consent to participate in this study.

## Author contributions

SO: Conceptualization, Data curation, Formal analysis, Funding acquisition, Investigation, Methodology, Project administration, Resources, Software, Supervision, Validation, Visualization, Writing – original draft, Writing – review & editing. GG: Conceptualization, Data curation, Formal analysis, Investigation, Methodology, Visualization, Writing – original draft, Writing – review & editing. ES-O: Data curation, Methodology, Software, Visualization, Writing – review & editing. EC: Conceptualization, Data curation, Formal analysis, Methodology, Writing – review & editing. ME: Conceptualization, Methodology, Writing – review & editing. LL: Writing – review & editing. AB: Resources, Supervision, Writing – review & editing. AW: Resources, Supervision, Writing – review & editing. AD: Methodology, Software, Supervision, Writing – review & editing. FR: Conceptualization, Data curation, Investigation, Methodology, Software, Validation, Visualization, Writing – review & editing. DN: Funding acquisition, Methodology, Resources, Supervision, Writing – review & editing. MD: Conceptualization, Data curation, Formal analysis, Funding acquisition, Investigation, Methodology, Resources, Software, Supervision, Validation, Visualization, Writing – review & editing.
