# Supplementary material for: Progressive remodeling of structural networks following surgery for operculo-insular epilepsy
Source: Front Neurol. 2024 Jul 31;15:1400601. doi: 10.3389/fneur.2024.1400601 (PMC11322451; doi:10.3389/fneur.2024.1400601)
Supplement: Supplementary file 1 [file Presentation_1.pdf]

Supplementary figures

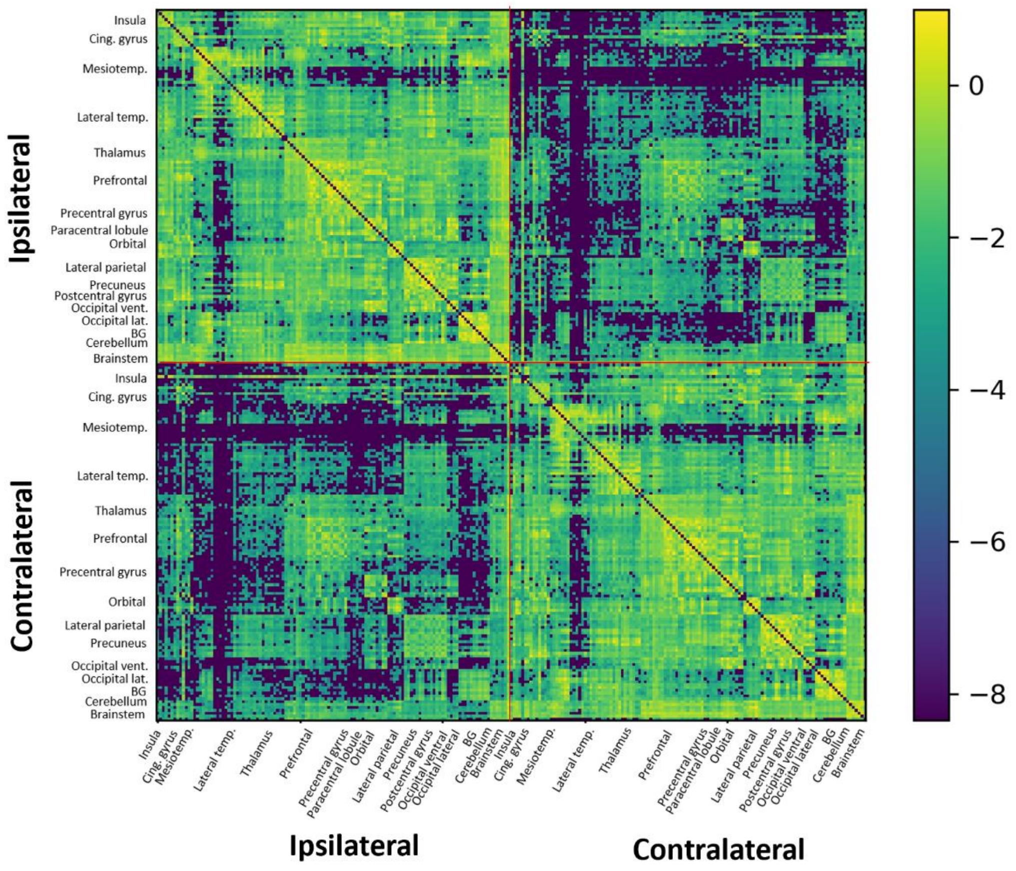

Figure S1. Average whole-brain COMMIT-weighted connectivity matrix before surgery (t_0). The average matrix was calculated from the preoperative matrices of all patients. For illustrative purposes, the log of the COMMIT weight was used. The colour bar corresponds to the log of the measured COMMIT weight.

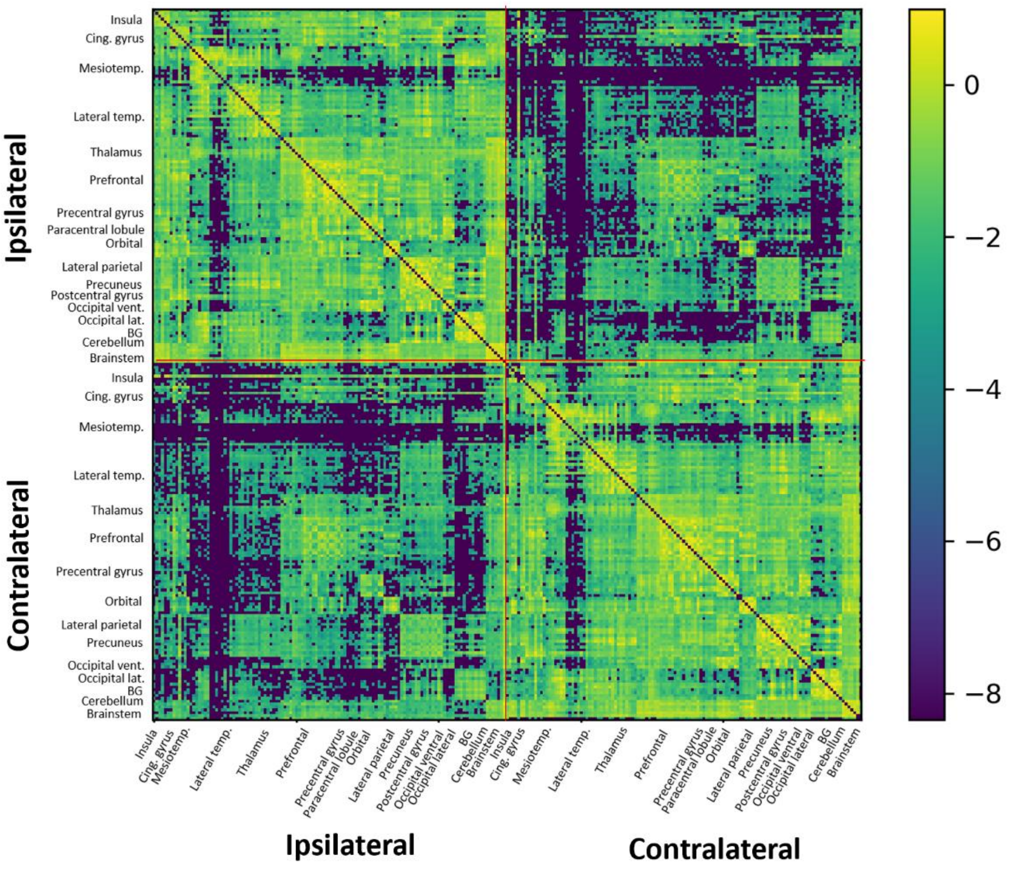

Figure S2. Average whole-brain COMMIT-weighted connectivity matrix 6 months following surgery (t_1). The average matrix was calculated from the 6-month postoperative matrices of all patients. For illustrative purposes, the log of the COMMIT weight was used. The colour bar corresponds to the log of the measured COMMIT weight.

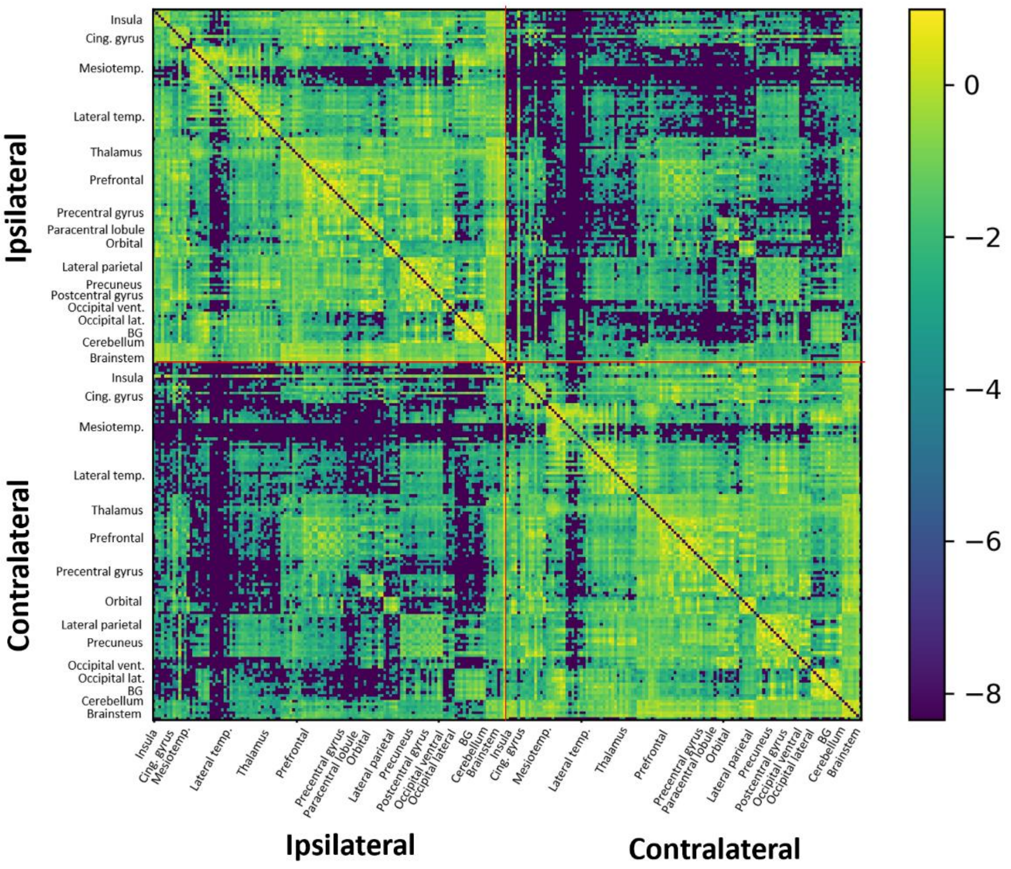

Figure S3. Average whole-brain COMMIT-weighted connectivity matrix 12 months following surgery (t_1). The average matrix was calculated from the 12-month postoperative matrices of all patients. For illustrative purposes, the log of the COMMIT weight was used. The colour bar corresponds to the log of the measured COMMIT weight.
