# Supplementary material for: Progressive remodeling of structural networks following surgery for operculo-insular epilepsy
Source: Front Neurol. 2024 Jul 31;15:1400601. doi: 10.3389/fneur.2024.1400601 (PMC11322451; doi:10.3389/fneur.2024.1400601)
Supplement: Supplementary file 2 [file Table_1.docx]

| **Graph theoretic measures** | **Definition** |
| --- | --- |
| Nodal measures |  |
| Betweenness centrality | Number of shortest paths that pass through a node. Reflects the level of integration of a node. |
| Clustering coefficient | Fraction of connected triangles around a node. Reflects the degree of cliquishness of a node. |
| Local efficiency | Average of the inverse shortest path length in the neighbourhood a node. Correlates with clustering coefficient and reflects the degree cliquishness of a node. |
| Nodal Strength | Sum of the weights of links connected to the node. Reflects the extent to which a node is relevant to the graph. |
| Global measures |  |
| Characteristic path length | Average of the shortest path length across all nodes. Inversely related to the ease of information flow. |
| Global efficiency | Inverse of the characteristic path length. Related to the ease of information flow. |
| Small-worldness | Ratio of average clustering coefficient to characteristic path length. Reflects the balance between segregation and integration. |
| Average betweenness centrality | Betweenness centrality averaged across all nodes |
| Average clustering coefficient | Clustering coefficient averaged across all nodes |
| Average nodal strength | Nodal strength averaged across all nodes |

**Table S1.** Definitions of the graph theoretic metrics investigated in this study (1–7).

|  | **Early postoperative changes (t_0_ vs t_1_)** | | | **Late postoperative changes (t_1_ vs t_2_)** | | |
| --- | --- | --- | --- | --- | --- | --- |
| **Postoperative increase in connectivity strength** | Ipsilateral hippocampus (rostral portion) | - | Ipsilateral PHG | Contralateral MFG (dorsal portion) | - | Contralateral SPL (intraparietal portion) |
|  | Ipsilateral precentral gyrus (caudal dorsolateral portion) | - | Ipsilateral dorsal dysgranular insula |  |  |  |
|  | Contralateral fusiform gyrus | - | Contralateral occipital pole |  |  |  |
| **Postoperative reduction in connectivity strength** | Ipsilateral medial SFG | - | Ipsilateral ITG (caudolateral portion) | Ipsilateral IPL (caudal portion) | - | Ipsilateral IPL (rostrocaudal portion) |
|  | Ipsilateral OF cortex (orbital portion) | - | Ipsilateral inferior occipital gyrus | Contralateral MFG (ventrolateral portion) | - | Contralateral lateral occipital cortex (V5_MT) |
|  | Ipsilateral OF cortex (orbital portion) | - | Ipsilateral ITG (intermediate lateral portion) | Contralateral medial OF gyrus (area 13) | - | Contralateral cingulate gyrus (dorsal portion) |
|  | Ipsilateral OF cortex (lateral portion) | - | Ipsilateral IPL |  |  |  |
|  | Ipsilateral OF cortex (lateral portion) | - | Ipsilateral inferior occipital gyrus |  |  |  |
|  | Ipsilateral OF cortex (medial portion) | - | Ipsilateral lingual gyrus (caudal portion) |  |  |  |
|  | Ipsilateral OF cortex (lateral portion) | - | Ipsilateral ventral dysgranular and granular insula |  |  |  |
|  | Ipsilateral precentral gyrus (caudal dorsolateral portion) | - | Ipsilateral globus pallidus |  |  |  |
|  | Ipsilateral precentral gyrus (region of the tongue and larynx) | - | Ipsilateral caudate nucleus (dorsal portion) |  |  |  |
|  | Ipsilateral STG (area 41-42) | - | Ipsilateral globus pallidus |  |  |  |
|  | Ipsilateral medial SFG | - | Ipsilateral hypergranular insula |  |  |  |
|  | Ipsilateral IPL (caudal portion) | - | Ipsilateral lateral MFG |  |  |  |
|  | Ipsilateral dorsal MFG | - | Contralateral hypergranular insula |  |  |  |
|  | Ipsilateral thalamus (lateral prefrontal portion) | - | Contralateral medial SFG |  |  |  |
|  | Ipsilateral cuneus (rostral portion) | - | Contralateral superior occipital gyrus (lateral portion) |  |  |  |
|  | Contralateral lateral STG | - | Contralateral caudal hippocampus |  |  |  |
|  | Contralateral frontal operculum | - | Contralateral STG (area 41-42) |  |  |  |
|  |  |  |  |  |  |  |

**Table S2**. Links showing postoperative changes in COMMIT weights. Results of comparisons between t_0_ and t_1_ matrices (early postoperative changes) as well as between t_1_ and t_2_ matrices (late postoperative changes) are shown. Comparisons were performed using paired sample *t*-tests. Significance was thresholded at *p* ≤ 0.001 uncorrected. PHG = parahippocampal gyrus; MFG = middle frontal gyrus; OF = orbitofrontal; SPL= superior parietal lobule; SFG = superior frontal gyrus; ITG = inferior temporal gyrus; IPL = inferior parietal lobule; MFG = middle frontal gyrus; STG = superior temporal gyrus.

|  | **Early postoperative changes (t_0_ vs t_1_)** | | | | **Late postoperative changes (t_1_ vs t_2_)** | | | |
| --- | --- | --- | --- | --- | --- | --- | --- | --- |
|  | BC | CC | LE | NS | BC | CC | LE | NS |
| Whole-brain network | Increase in the contralateral postcentral gyrus (trunk area)  Decrease in the ipsilateral medial SFG | ND | ND | Decrease in the ipsilateral ITG (caudodorsal portion) and thalamus (posterior parietal and occipital portions) | ND | Increase in the contralateral OF cortex (lateral portion) | Increase in the contralateral OF cortex (lateral portion) | ND |
| Ipsilateral hemisphere subnetwork | ND | Increase in the medial SFG | ND | Decrease in the frontal operculum and thalamus (occipital portion) | Increase in the medial precuneus | ND | ND | ND |
| Contralateral hemisphere subnetwork | Increase in the OF cortex (lateral portion) | Decrease in the fusiform gyrus (mediodorsal portion) | Decrease in the fusiform gyrus (mediodorsal portion) | Increase in the cingulate gyrus (pregenual portion) | Decrease in the ITG (rostral portion) and lingual gyrus (rostral portion) | ND | ND | ND |
| Contralateral insular subnetwork | ND | ND | ND | ND | ND | ND | ND | ND |

**Table S3**. Nodes showing postoperative changes in regional graph theoretic measures. Analyses were performed on whole-brain networks and on sub-networks consisting of square matrices linking 1) ipsilateral regions to each other (ipsilateral hemisphere subnetwork), ii) contralateral regions to each other (contralateral hemisphere subnetwork) and iii) contralateral insular regions to each other (contralateral insular subnetwork). Results of network comparisons between t_0_ and t_1_ (early postoperative changes) as well as between t_1_ and t_2_ (late postoperative changes) are shown. Comparisons were performed using paired sample *t*-tests. Significance was thresholded at *p* ≤ 0.001 uncorrected. BC = betweenness centrality; CC = clustering coefficient; LE = local efficiency; NS = nodal strength; SFG = superior frontal gyrus; OF = orbitofrontal; ITG = inferior temporal gyrus.

ND = none of the nodal comparisons revealed statistically significant differences.

**Supplemental references**

1. Bonilha L, Nesland T, Martz GU, Joseph JE, Spampinato M V., Edwards JC, et al. Medial temporal lobe epilepsy is associated with neuronal fibre loss and paradoxical increase in structural connectivity of limbic structures. Journal of Neurology, Neurosurgery and Psychiatry. 2012;83(9):903–9.

2. Bassett DS, Bullmore E. Small-world brain networks. Neuroscientist. 2006;12(6):512–23.

3. Bullmore E, Sporns O. Complex brain networks: Graph theoretical analysis of structural and functional systems. Nature Reviews Neuroscience. 2009;10(3):186–98.

4. Sporns O. Graph theory methods: Applications in brain networks. Dialogues in Clinical Neuroscience. 2018;20(2):111–20.

5. da Silva NM, Forsyth R, McEvoy A, Miserocchi A, de Tisi J, Vos SB, et al. Network reorganisation following anterior temporal lobe resection and relation with post-surgery seizure relapse: A longitudinal study. Neuroimage Clin. 2020;27.

6. Ji GJ, Zhang Z, Xu Q, Wei W, Wang J, Wang Z, et al. Connectome reorganization associated with surgical outcome in temporal lobe epilepsy. Medicine (United States). 2015;94(40):1–9.

7. Obaid S, Rheault F, Edde M, Guberman GI, St-Onge E, Sidhu J, et al. Structural connectivity alterations in operculo-insular epilepsy. Brain Sciences. 2021;11(8).
